# An Assessment of H1N1 Influenza-Associated Acute Respiratory Distress Syndrome Severity after Adjustment for Treatment Characteristics

**DOI:** 10.1371/journal.pone.0018166

**Published:** 2011-03-25

**Authors:** Brent P. Riscili, Tyler B. Anderson, Hallie C. Prescott, Matthew C. Exline, Madhuri M. Sopirala, Gary S. Phillips, Naeem A. Ali

**Affiliations:** 1 The Division of Pulmonary, Allergy, Critical Care and Sleep Medicine, The Ohio State University Medical Center, Columbus, Ohio, United States of America; 2 The Division of Infectious Diseases, The Ohio State University Medical Center, Columbus, Ohio, United States of America; 3 Department of Internal Medicine, The Ohio State University Medical Center, Columbus, Ohio, United States of America; 4 Center for Biostatistics, The Ohio State University Medical Center, Columbus, Ohio, United States of America; University of Giessen Lung Center, Germany

## Abstract

Pandemic influenza caused significant increases in healthcare utilization across several continents including the use of high-intensity rescue therapies like extracorporeal membrane oxygenation (ECMO) or high-frequency oscillatory ventilation (HFOV). The severity of illness observed with pandemic influenza in 2009 strained healthcare resources. Because lung injury in ARDS can be influenced by daily management and multiple organ failure, we performed a retrospective cohort study to understand the severity of H1N1 associated ARDS after adjustment for treatment. Sixty subjects were identified in our hospital with ARDS from “direct injury” within 24 hours of ICU admission over a three month period. Twenty-three subjects (38.3%) were positive for H1N1 within 72 hours of hospitalization. These cases of H1N1-associated ARDS were compared to non-H1N1 associated ARDS patients. Subjects with H1N1-associated ARDS were younger and more likely to have a higher body mass index (BMI), present more rapidly and have worse oxygenation. Severity of illness (SOFA score) was directly related to worse oxygenation. Management was similar between the two groups on the day of admission and subsequent five days with respect to tidal volumes used, fluid balance and transfusion practices. There was, however, more frequent use of “rescue” therapy like prone ventilation, HFOV or ECMO in H1N1 patients. First morning set tidal volumes and BMI were significantly associated with increased severity of lung injury (Lung injury score, LIS) at presentation and over time while prior prescription of statins was protective. After assessment of the effect of these co-interventions LIS was significantly higher in H1N1 patients. Patients with pandemic influenza-associated ARDS had higher LIS both at presentation and over the course of the first six days of treatment when compared to non-H1N1 associated ARDS controls. The difference in LIS persisted over the duration of observation in patients with H1N1 possibly explaining the increased duration of mechanical ventilation.

## Introduction

Pandemic influenza drove significant increases in healthcare utilization across several continents in 2009.[1], [2], [3], [4], [5] Many of these patients were critically ill with need for significant ventilatory support, mechanical ventilation and even extracorporeal support.[6] In fact, despite the lack of systematic data available it has been suggested that influenza associated ARDS, because of its severity may be considered a preferred condition for extracorporeal support when severe.[7] Several other factors have been observed in cases of H1N1 associated respiratory failure. Female gender, obesity and younger age appear particularly over-represented amongst those patients with H1N1.[4], [8], [9] In addition, associated organ failure with shock and renal failure appeared to be very common in these same patients.

Unfortunately, these factors can be associated with worsening of hypoxemia or ARDS severity. Obesity's effects on normal pulmonary physiology may predispose to the development of ALI, but also worsen its manifestation [10] although its effect on outcome is controversial. [11] Separately, it is possible that additional organ failures like shock and renal failure can independently influence the severity of acute lung injury. Fluid accumulation whether secondary to renal failure or fluid resuscitation can worsen oxygenation and thus lung injury.[12] In addition, other associated treatments like tidal volumes used and transfusion practices could influence the severity or duration of ALI in critically ill patients.[13], [14] In fact, if the severity of influenza associated acute lung injury is higher than typical ARDS, the use of low-tidal volume ventilation may be abandoned because of its adverse effects on oxygenation.[13]

As a result, we hypothesized that the severity of the acute respiratory distress syndrome in H1N1 patients was at least partly explained by patient or treatment characteristics. No prior studies have directly compared H1N1- associated ARDS with contemporary ARDS control patients. Therefore, we performed a retrospective cohort analysis comparing patients with ARDS in association with H1N1 or other non-H1N1 causes of direct lung injury. We specifically included an assessment of patient characteristics, ventilator strategies, transfusion and fluid balance practices. The purpose of this study was to better understand the interaction between associated treatments and ARDS severity in order to put in context the importance H1N1 influenza as a specific cause.

## Methods

### Objectives

We performed a retrospective analysis of prospectively identified patients with ARDS admitted to the Medical Intensive Care Unit over a three month time period from October 1^st^ until December 31^st^, 2009 at The Ohio State University Medical Center. The primary goal was to describe the severity of illness over time in patients with H1N1 associated ARDS in order to determine whether treatments for ARDS or associated organ failure could explain the prolonged and intense course of critical illness in these patients. We specifically hypothesized that associated organ failure (like shock) would lead to specific treatments (like transfusions) that would contribute to the severity of ARDS.

### Participants

From October 1^st^ until December 31^st^, 2009, our ICU had standardized protocols in place to obtain nasopharyngeal swabs from all patients admitted to the ICU with respiratory failure to screen for influenza. Patients requiring mechanical ventilation were screened daily for hypoxemia consistent with acute lung injury (PaO2/FiO2 ratio <300) according to consensus criteria.[15] Physician investigators then reviewed the case for radiographic and clinical characteristics in order to confirm the suspicion that hypoxemia was caused by ARDS (PaO2/FiO2 ratio <200).[15] Subjects were only excluded from study if they were younger than eighteen or a prisoner or ward of the state. To minimize potential confounders associated with systemic infections and trauma this cohort was confined to those subjects with presumed “direct” lung injury.

### Description of Procedures or Investigations undertaken

Cases were identified as being H1N1-associated if nasal swab or respiratory secretions were positive for novel influenza A (H1N1) by specific rapid antigen or culture testing. Non-H1N1 associated cases were the remaining cases identified. H1N1 positive patients without ARDS are not included.

Nasal swabs that were processed for confirmatory influenza testing by polymerase chain reaction testing were confirmed by panel (xTAG) or specific influenza A and B PCR (Luminex Molecular Diagnostics, Toronto, CA).[16] Positive tests were confirmed as being caused by H1N1 using the Prodesse ProFlu-ST, Influenza A [2009] real-time PCR (H1N1 subtyping) assay (Focus Diagnostics, Cypress, CA). Not all samples were processed for PCR confirmation. However, in our central lab all samples processed clinically or automatically after random selection (n = 10 per month in non-subtyped samples) were 2009 H1N1 positive during the observation period.

After identifying patients as having ARDS, charts were reviewed for demographic and severity of illness information. Special attention was paid to identifying the duration of symptoms, clinical factors and treatments delivered over the first six days of mechanical ventilation. The six days of follow-up was based on our prior experience of the median duration of mechanical ventilation in our institution and a concern that after this point hypoxemia would be confounded by too many other variables to allow interpretation. This was collected from admitting histories or notes. Specifically, the daily ventilator settings, pulmonary compliance and oxygenation parameters were recorded from first available values after eight am each morning. In addition, fluid balance, transfusions and the presence of shock were recorded on a daily basis. ARDS severity was primarily measured as the lung injury (Murray) score.[17]

Cohort demographics, co-morbidities and cause of ARDS were collected by manual chart review. Medical history was categorized according to notation in the admitting history available for all patients at the time of the ICU admission. Cause of ARDS was obtained from information available in the same admitting history. Severity of illness was calculated as the SOFA score [18], [19] and ARDS severity as PaO2:FiO2 ratio and the lung injury (Murray) score.[17] To allow the comparison of LIS between groups with different durations of ventilator use, we also present LIS as an area under the curve (AUC) per day of measurement where specified. Covariates collected over the first 6 days of ventilator care were the following: highest tidal volume used for at least one hour during the observation day, net fluid balance, transfusion of any blood product, time to antiviral therapy, shock and vasopressor use.

### Ethics

Review of these records and the performance of this study were approved in advance by our local human subject's protection committee. (2009E0979) The Ohio State University Medical Center's human subjects Institutional Review Board approved this research as not needing informed consent as all data was collected without identifying PHI and analyzed anonymously.

### Statistical Methods

Cohort characteristics were quantified and compared using Student's t-test or Wilcoxon rank sum test for all quantitative measures depending on the normality of the data. Dichotomous variables were assessed for significant difference using the Pearson's chi-square statistic or Fisher's exact test. For analysis of the factors associated with ARDS severity over time, random-effects maximum likelihood linear regression was used with H1N1 status as the primary risk factor. Random-effects regression was used since observations were nested within subject over time, thus the within and between subject variability was used to estimate the standard errors used to test the model coefficients. Baseline and treatment related covariates were then added into the model individually and any variable changing the risk factor coefficient by ±15% was included in the final adjusted analysis. Variables specifically tested included, gender, BMI, age, race, transfusion days, net fluid balance, vasopressor use and cause of lung injury.

## Results

**Figure 1:**
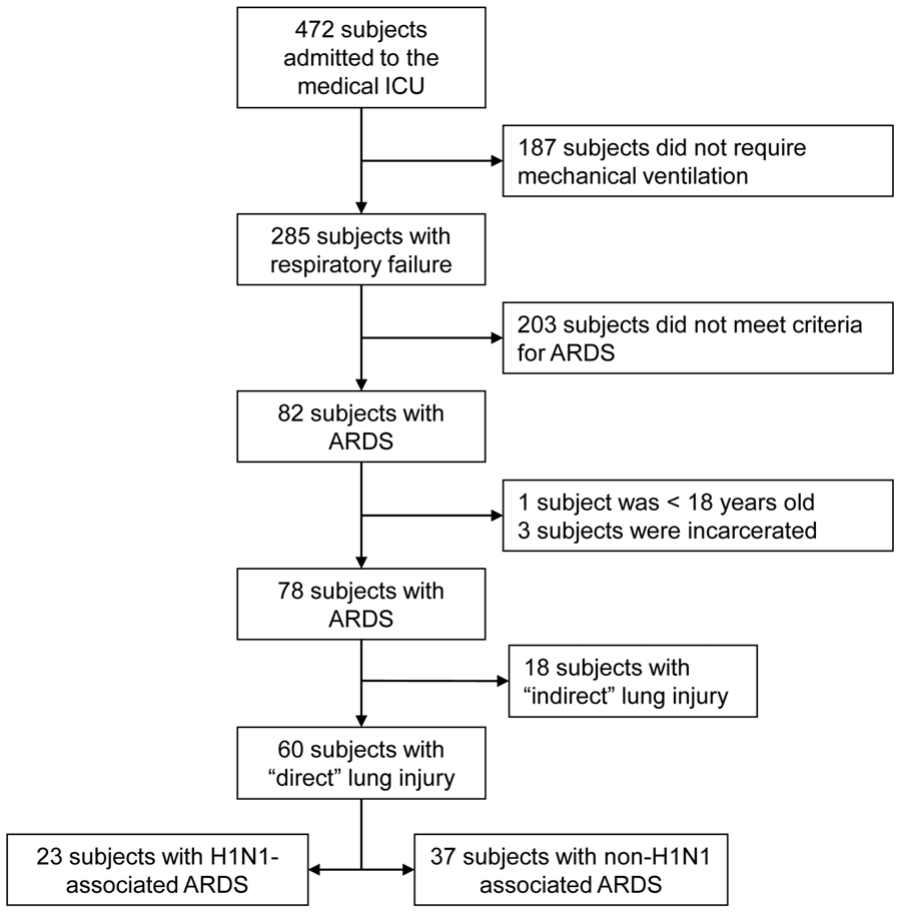
Cohort enrollment diagram.

Eighty-two patients (17.4% of total) were admitted to the medical ICU with a diagnosis of ARDS. (Figure 1) Of 60 patients with direct lung injury, 23 (38.3%) had influenza-associated disease (14/23 or 60.8% confirmed H1N1 by PCR) although all tested cases presenting to our institution from September 2009 through April 2010 were found to be H1N1. Similar to other prior reports, subjects with H1N1-associated ARDS were slightly younger, with a larger body mass index. Additionally, these subjects were more likely to receive mechanical ventilation sooner after the onset of symptoms. (Table 1)

**Table 1:** Baseline characteristics of the ARDS cohort with direct lung injury.

	H1N1-associated ARDS (n = 23)	Non H1N1- associated ARDS (n = 37)	Total cohort (n = 60)	*p*-value^1^
Age, years	42.5±16.25	50.9±26.7	47.7±23.5	0.177
Male (%)	51.4	47.8	50.0	>0.99
BMI, median (IQR)	35.2 (27.5–50.8)	30.7 (24.0–36.6)	32.3 (25.1–41.8)	0.064
Pregnancy, n (% of females)	2 (16.7)	0 (0)	2 (6.7)	0.152
Caucasian (%)	73.9	70.3	71.7	>0.99
Hispanic (%)	34.8	24.3	28.3	0.396
Medical History, n (%)				
• Asthma	2 (8.7)	4 (10.8)	6 (10.0)	>0.99
• COPD	1 (4.4)	8 (21.6)	9 (15.0)	0.134
• Cancer history	1 (4.4)	8 (21.6)	9 (15.0)	0.134
• DM	4 (17.4)	7 (18.9)	11 (18.3)	>0.99
• Immunocompromised condition	2 (8.7)	5 (13.5)	7 (11.7)	0.697
Tobacco use, n (%)	7 (30.4)	9 (24.3)	16 (26.7)	0.60
Prior statin use, n (%)	3 (13.0)	13 (35.1)	16 (26.7)	0.06
Influenza testing				
• Nasopharyngeal swab, n (%)	15/24 (62.5)	0/54 (0.0)		
• Bronchoalveolar lavage fluid, n (%)	10/11 (90.1)	NR		
Duration of symptoms before hospitalization (hrs), median (IQR)	72 (48–168)	72 (24–120)	72 (36–156)	0.407
Time until need for mechanical ventilation (hrs), median (IQR)	0 (0–0)	0 (0–1)	0 (0–0)	0.084
Shock (%)	56.5	56.8	56.7	>0.99
Cardiovascular failure, median (IQR)				
• Days of pressor use through day 3	2 (0–3)	1 (0–3)	1 (0–2)	0.126
• Days of pressor use through Day 6	2 (0–3)	1 (0–3)	1 (0–3)	0.197
• Average CV SOFA score through Day 3	3 (1–4)	1.7 (0.3–3)	1.7 (0.7–3.3)	0.060
• Average CV SOFA score through day 6	2.0 (0.5–3.2)	1.7 (0.3–2.8)	1.7 (0.4–3)	0.197
CXR score (0–4 quadrants with infiltrates) through day 6, median (IQR)	4 (4–4)	4 (3–4)	4 (4–4)	0.003
SOFA score through day 6	11.9±4.7	9.5±5.5	10.5±5.3	0.002
SOFA (no respiratory subscore) through day 6	8.5±4.4	6.9±5.0	7.6±4.8	0.006
PaO2:FiO2 through day 6, median (IQR)	101.5 (65–134)	148 (95–190)	119 (74–170)	<0.001
Lung Injury (Murray) Score, median (IQR)	3.5 (3.0–3.8)	2.8 (2.5–3.3)	3 (2.5–3.5)	<0.001

All values represent means ± SD unless otherwise specified.

1 *p*-values based on the following tests: *t-*test for variables presented as means, Wilcoxon rank-sum test for variables presented as the median, Fisher's exact test of association for categorical variables.

Subjects with H1N1-associated ARDS also had significantly higher severity of illness and worse oxygenation at presentation when compared to other ARDS patients. (Table 1) Although there was slightly more hypotension as measured by the SOFA cardiovascular sub score over the days of observation (Table 2) there were no significant differences in shock or vasopressor requirement over the first six days. In addition, there were no significant differences in tidal volumes or fluid balance over the observation period. (Table 2) However, there was a trend toward a higher frequency of transfusion over the course of the first six days of treatment that did not reach a statistically significant level. (Table 2) Oseltamivir was administered to all H1N1 subjects at a dose of 150 mg twice daily within 2 days of the onset of ILI symptoms except one patient who had renal failure and received 75 mg BID. A further two subjects received this level after two days of 75 mg twice daily.

**Table 2:** Treatment variables over the first six days of therapy and outcomes.

	H1N1-associated ARDS (n = 23)	Non-H1N1 associated ARDS (n = 37)	Total cohort (n = 60)	*p*-value^1^
Tidal Volume(ml/kg PBW), median (IQR)				
• Day 1	6.1 (6.0–6.8)	6.5 (6.5–6.8)	6.5 (6.0–7.2)	0.789
• Day 3	5.7 (5.7–6.1)	6.0 (6.0–6.2)	6.1 (6.0–6.8)	0.309
• Average over first 6 days	5.9 (5.9–6.0)	6.4 (6.1–6.5)	6.3 (6.0–6.5)	<0.001
Transfusions				
• Prior to ICU admit (%)	34.8	21.6	26.7	0.369
• Any transfusion to Day 6 (%)	56.5	43.2	48.3	0.427
• Days with any transfusion to Day 6, median (IQR)	1 (0–3)	0 (0–2)	1 (0–2)	<0.001
• Proportion of first 6 ICU days with transfusion, median (IQR)	20 (0–60)	0 (0–50)	0 (0–50)	0.390
Vasopressor use				
• Total days of therapy, median (IQR)	2 (0–5)	1 (0–4)	2 (0–5)	0.411
• To Day 3, (%)	65.2	59.5	61.7	0.787
Net fluid balance (mean, ±SD)				
• to Day 6	9,075±8,070	8,063±8,684	8,451±8,399	0.654
• On days with MAP>70 only	1,855±4,184	1,332±3,768	1,552±3,888	0.718
ECMO use (%)	17.4	0.0	6.7	0.018
HFOV use (%)	26.1	5.4	13.3	0.045
Antiviral therapy				
• Antiviral therapy initiated (%)	23 (100.0)			
• Days from hospital admit until treatment, median (IQR)	0 (0–1)			
ICU length of stay (d), median (IQR)	20 (9–31)	11 (6–17)	15 (7–23)	<0.001
Ventilator days, median (IQR)	20 (8–31)	10 (4–16)	12.5 (6–21)	<0.001
Ventilator-free days to day 30, median (IQR)	0 (0–17)	7 (0–26)	0 (0–23)	<0.001
Hospital length of stay (d), median (IQR)	25 (14–33)	18 (12–24)	20 (12–29)	<0.001
Hospital free days to day 60 (d), median (IQR)	22 (0–40)	30 (0–45)	27 (0–43.5)	0.055
ICU survival (%)	63.0	62.1	62.5	0.899
Hospital survival (%)	63.0	62.1	62.5	0.899

All values represent means ± SD unless otherwise specified.

1 *p*-values based on the following tests: *t-*test for variables presented as means, Wilcoxon rank-sum test for variables presented as the median, Fisher's exact test of association for categorical variables.

**Figure 2:**
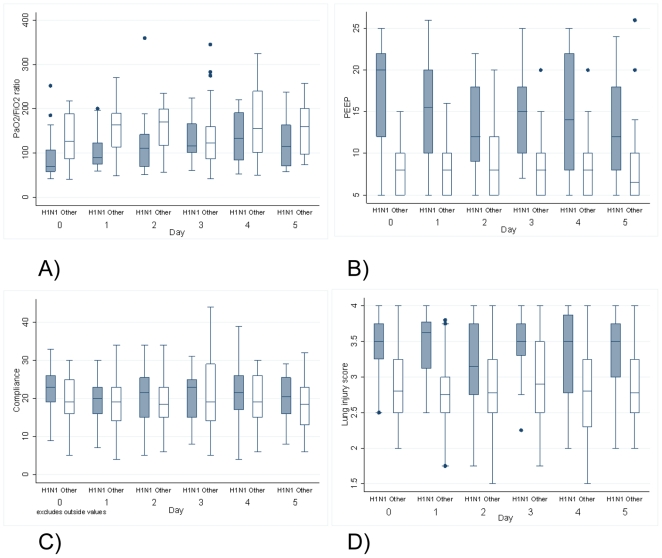
ARDS-related pulmonary measures over the first six days of treatment. All variables [A) PaO_2_/FiO_2_ ratio; B) positive end expiratory pressure; C) pulmonary compliance; D) lung injury (Murray) score] are recorded from the first complete data point after 8am on each day of observation while on conventional mechanical ventilation. Values from subjects requiring ECMO were dropped on days this modality was utilized. Bars represent box plots for each variable by day and cause of ARDS (H1N1-associated vs non H1N1). Center line represents the median; edges of each box represent the 75^th^ and 25^th^ percentiles and whiskers the maximum and minimum values. Dots indicate outliers. (n, for H1N1 associated ARDS is 23, 20, 18, 17, 16 and 14 and non-H1N1 is 30, 33, 34, 30, 29 and 22 for day 0–5).

The level of positive end-expiratory pressure appeared to be higher in patients with H1N1-associated ARDS in keeping with its associated worsened hypoxemia. (Figure 2 and Table 1) Consistent with prior observations, there was also more frequent use of rescue therapies like extracorporeal membrane oxygenation or high frequency oscillatory ventilation in H1N1-associated ARDS, although these interventions were not protocolized and therefore cannot directly reflect acuity. (Table 2)

Lung injury scores (LIS) were significantly worse in patients with H1N1-associated ARDS. (Table 3) Assessment of variables associated with lung injury severity revealed that variables that significantly changed the relationship between H1N1 status and LIS were BMI, evidence of statin use at the time of admission and daily tidal volume delivered. Tidal volume and BMI were directly related and significantly associated with a worsening of lung injury; whereas, statin use was associated with lower LIS. After adjustment for each of these factors, both the daily and total AUC for LIS was significantly higher in patients with H1N1 associated disease. (Table 4) Adjustment for transfusions, fluid balance or shock treatment had no direct influence on the relationship between LIS and H1N1 status. In addition, there was no evidence of association between time to influenza virus-active antiviral administration (Oseltamivir) in patients with H1N1 and severity of LIS at presentation or AUC LIS.

**Table 3:** Unadjusted lung injury score by influenza status.

	H1N1-associated ARDS	Non H1N1-associated ARDS	P value
**Day 0 LIS, table-2-caption(mean ± SD)**	3.24±0.83	2.84±0.45	0.019
**AUC LIS/day of observation, (mean ± SD)**	3.17±0.69	2.71±0.57	0.007

AUC, area under the curve for first daily lung injury scores.

**Table 4:** Adjusted lung injury score by influenza status.

	H1N1-associated ARDS		Non H1N1-associated ARDS		P value
LIS	Mean	95% CI	Mean	95% CI	
**Day 0**	3.20	3.17–3.53	3.03	3.35–2.86	0.006
**Day 1**	3.16	3.15–3.49	3.00	3.32–2.84	
**Day 2**	3.13	3.13–3.46	2.98	3.29–2.82	
**Day 3**	3.11	3.10–3.43	2.95	3.27–2.79	
**Day 4**	3.09	3.06–3.42	2.92	3.24–2.75	
**Day 5**	3.08	3.02–3.40	2.90	3.21–2.71	
**AUC LIS**	14.1	12.0–16.1	8.2	6.9–9.5	<0.001

Analyses adjusted for significant covariates (change of risk factor coefficient by ±15%) which included BMI, statin use and daily tidal volume used in addition to age and SOFA score.

## Discussion

We present the first cohort study that directly compares the severity of H1N1-associated ARDS to a contemporaneous cohort of non influenza associated ARDS. The use of ARDS control subjects and the depth and breadth of co-interventions described make this analysis unique. Our data quantitatively confirm what was suggested by observational cohort studies in clinical practice.[2], [3], [4] By using a very specific control group and including a rigorous collection of treatment practices, we can be more confident in our conclusion that the severity of H1N1 associated ARDS was higher than non-H1N1 associated lung injury. As such, it may justify a designation as a unique phenotype of ARDS that warrants specific description.

Several observations are interesting in this cohort. First of all, despite the impression of multiple organ failure in H1N1-associated illness, the severity of illness at presentation in H1N1 associated patients was largely driven by more severe hypoxemia. Additionally, this difference in severity of lung injury appears to extend for several days beyond presentation. This is supported by several pathologic or autopsy studies that have demonstrated very severe diffuse alveolar damage in H1N1 patients.[20], [21] Because of the collection of interventions, our data suggests that it is unlikely that treatment-related lung injury played a role in propagating hypoxemia as tidal volumes and other practices were similar in both treatment groups. Therefore, the severity of pulmonary injury is not likely related to co-interventions, but the direct virulence of the pathogen or immune response of the host.[22], [23] The data from this study can be reasonably used to suggest that institutions should develop well-defined approaches to care for severe influenza associated ARDS patients, because of the severity of hypoxemia at onset. In addition, the duration of critical illness and severity of hypoxemia confirmed in our cohort in comparison to more “routine” ARDS confirms that any allocated resources will be utilized for significant periods of time.

The second observation of interest is that when compared to contemporaneous control ARDS patients, the severity of non-pulmonary organ failure was no worse in H1N1-associated disease. In fact, there were no major differences in any non-pulmonary organ failures between the two groups. One organ failure that is very relevant to the management of refractory ARDS is shock. The presence of shock could prevent the use of conservative fluid management strategy which reduces hypoxemia and ventilator use or make the use of “rescue” therapies like prone ventilation or HFOV more difficult. However, while there were numerically small increases in the days of vasopressor use, transfusions and net fluid balance on days without hypotension in H1N1 patients, these factors did not appreciably influence the severity of lung injury in our analysis. The finding of equivalent non-pulmonary organ failure could be related to the small size of our cohort or how severely ill our control ARDS subjects were. In fact, 57.1% of our non-H1N1 ARDS patients had evidence of shock at presentation, whereas only ∼32% of patients in published series have this significant organ failure.[12], [24] Our analysis suggests that this organ failure is likely no more common than in other patients with ARDS and is simply part of “expected” multiple organ failure. However, our cohort of non-H1N1 ARDS patients may be particularly ill.

### Limitations

There are several limitations to our study. First, our sample is relatively small and derived from a single center. It is possible that single center management can be confounded by unmeasured co-interventions and variation in practice. To counteract this effect, we have gone to great lengths to collect treatment variables that are likely to impact overall oxygenation in ARDS patients. Tidal volumes and fluid management strategies are both known to influence oxygenation [12], [13] in ARDS patients and transfusions to influence its development. [14] It was our aim to include these variables to limit the number of potential unmeasured covariates which would effect our conclusions. Another difference in our study is the use of lung injury score to measure severity of ARDS. The use of the lung injury score as a measure of ARDS severity is not routine. This was chosen primarily, because it has been suggested as a tool to identify patients who may need rescue therapy,[17] but also because it assesses more than simply oxygenation. As such it could have been more sensitive to changes other than hypoxemia. As a result, we are more assured that our finding that the difference in severity of illness is related to simply worsened gas exchange impairment is true. However, similar trends in severity were seen in PaO2/FiO2 ratio and ventilator use. Still it would have been informative to collect data on other variables like oxygenation index. Additionally, it is possible that our findings are confounded by the relatively narrow range of management for these patients. Our conventional ventilator management is protocolized and may prevent the detection of greater effects in other units with a wider variation in practice. Rescue therapies, in contrast, are initiated at clinician discretion and it is possible that they were instituted more readily in H1N1 patients. The risk of this confounding our interpretation is mitigated by the use of a lung function outcome instead of vital outcome or ventilator use, but still raises some caution. Finally, it is unclear whether the same severity assessments would be true in non-pandemic influenza. Further research is likely needed to clarify this question and to determine whether this is related to other more important patient-centered outcomes like mortality.

### Conclusions

Despite our limitations, we believe this cohort study provides the first description of co-interventions likely to influence severity of lung injury in ARDS in H1N1 patients. The comparison of an H1N1 cohort with a more relevant reference group of ARDS patients, we feel ultimately makes the conclusions more robust when trying to assess why novel influenza stretched ICU resources this past year. It demonstrates that hypoxemia and lung injury in patients with H1N1 is due specifically to the extent of respiratory injury from the infection itself and not to secondary injury related to the treatment of associated multi-organ failure. Whether this is particular to H1N1 or generalized influenza associated ARDS is unclear. This information may be of use in preparations for future pandemics.
